# Current Recommendations for the Use of Sound Therapy in Adults with Hyperacusis: A Scoping Review

**DOI:** 10.3390/brainsci14080797

**Published:** 2024-08-09

**Authors:** Nighat Kalsoom, Kathryn Fackrell, Dayana El Nsouli, Hayley Carter

**Affiliations:** 1 Adult Audiology, Royal Derby Hospital, University Hospitals of Derby and Burton, Derby DE22 3NE, UK; 2 School of Health and Social Care, University of Lincoln, Brayford Pool, Lincoln LN6 7TS, UK; 3 NIHR Nottingham Biomedical Research Centre, Ropewalk House, 113 The Ropewalk, Nottingham NG1 5DU, UK; kathryn.fackrell@nottingham.ac.uk; 4 Hearing Sciences, Mental Health and Clinical Neuroscience, School of Medicine, University of Nottingham, Nottingham NG7 2UH, UK; 5 Pharmacy Department, Royal Derby Hospital, University Hospitals of Derby and Burton, Uttoxeter Road, Derby DE22 3NE, UK; dayana.elnsouli@nhs.net; 6 School of Health Sciences, University of Nottingham, University Park, Nottingham NG7 2RD, UK; 7 Musculoskeletal Outpatients, London Road Community Hospital, University Hospitals of Derby and Burton NHS Foundation Trust, Derby DE1 2QY, UK; hayley.carter1@nhs.net

**Keywords:** hyperacusis, sound intolerance, sound therapy, sound generators, sound interventions

## Abstract

Hyperacusis is a condition that is characterized by hypersensitivity to normal everyday sounds or reduced sound tolerance and can affect patients in distressing ways. Sound therapy is a treatment intervention that is used to desensitize patients. However, as yet, there is a lack of understanding on how it is used in clinical practice, the different types of devices, or how to use them. The aim of this scoping review was to establish the current use of sound therapy in adults with hyperacusis and identify any factors that may influence treatment. Methodology: An established methodological framework was used to formulate the research question and guide the search strategy and reporting. The inclusion criteria were studies reporting adult (>18 years) populations with hyperacusis and sound therapy treatments which were published in any language. Searches of electronic databases (CINAHL, Cochrane Library, Medline (EBSCO), Scopus, PsycINFO) identified 31 studies that met the inclusion criteria (completed in April 2024). Data from included records were collated and summarized descriptively.

## 1. Introduction

Hyperacusis is a ‘*reduced tolerance to sound (s) that are perceived as normal to the majority of the population or were perceived as normal to the person before their onset of hyperacusis*’, as defined by consensus by Adams and colleagues in 2021 [1]. Hyperacusis can co-exist with other types of sound sensitivities like phonophobia (sound sensitivity linked to a fear of sound and linked to migraine) and misophonia (causes a negative reaction (anger or rage) within an individual to certain human-generated sounds like breathing or eating) [2,3,4]. Most commonly, it is associated with tinnitus (the perception of a sound or sensation within the head or ear(s) [5], with up to 86% of patients experiencing hyperacusis symptoms as a primary complaint [6]. Other conditions/disorders that are also thought to exist with hyperacusis include autism spectrum disorder (ASD), Williams Syndrome, depression, post-traumatic stress disorder, Lyme disease, neurological conditions such as middle cerebral artery aneurysm, Multiple Sclerosis, fibromyalgia, and upper respiratory tract infections including SARS-CoV-2 [7,8,9,10,11]. 

A systematic review conducted by Ren and Colleagues in 2021 reported a hyperacusis prevalence of 0.2% to 17.2% in the general population worldwide, 3.8% to 67% in those with a special occupational background (such as musicians), and 4.7% to 95% in those with diseases/comorbidities linked to hyperacusis [12].

The physiological mechanisms for hyperacusis are not clearly defined. It has been proposed to be linked to peripheral hearing loss and a central model of pathogenesis termed the Neurophysiological model, where the autonomic nervous system and limbic system are activated by the auditory system [2,13,14]. Irrespective of the physiological mechanisms driving this complex condition, it is reported to impact upon an individual’s sleep, hearing, concentration and psychological well-being, thus negatively affecting their quality of life [7,15].

As there is no specific test for the diagnosis of hyperacusis, assessment involves self-report questionnaires and conducting interviews with specific questions around background, noise sensitivities, and other medical conditions of relevance [7]. Currently, no specific validated outcome measure for hyperacusis exists (in a hyperacusis population) [16]. However, a number of self-report questionnaires, such as the Hyperacusis questionnaire (HQ) [17], Geräuschüberempfindlichkeit (GÜF) [18], and Multiple Activity Scale for Hyperacusis [19], have been developed. More recent self-report questionnaires developed and being used for hyperacusis also include the Hyperacusis Impact Questionnaire (HIQ) and Sound Sensitivity Symptoms Questionnaire (SSSQ) [20]. Other self-report outcome measures are used to assess comorbidities including tinnitus or psychological well-being such as the Tinnitus Handicap Inventory [15] and the Hospital Anxiety and Depression Scale (HADS), respectively [7]. Physiological tests used for assessment include conducting subjective hearing tests (audiogram) and assessing Uncomfortable Loudness Levels (ULLs)/Loudness Discomfort Levels (LDLs) [7]. However, there is a difference in opinion regarding the format of assessment [20]. This is dependent on whether the Tinnitus Retraining Therapy (TRT) Protocol is implemented [21] or another assessment format is used [7]. The TRT protocol has a very specific format of interview questions [20] that are used for assessment of tinnitus and hyperacusis. Other assessment formats differ in their questioning/interviewing style and are less prescriptive, including what self-report outcome measures are recommended for use [7] and whether these can be used to monitor outcomes/improvements over time [16]. 

Currently, there is no consensus on the treatment for hyperacusis [1,7]. A number of interventions that have been reported in the literature include Cognitive Behavioural Therapy (CBT), counselling alone, Tinnitus Retraining Therapy (TRT)—which includes some directive counselling and sound therapy—surgery where appropriate, pharmacological therapy, and sound therapy using devices [1,7]. In clinical practice, sound therapy is often used as part of a treatment plan for hyperacusis. However, as yet, there are no recommended guidelines on what sound therapy to use or how to use it in the UK. There is no contemporary evidence for the use of sound therapy in adults with hyperacusis, which has been highlighted by the James Lind Alliance as a current research priority [15]. This study aims to support the development of evidence-based clinical guidelines by reviewing the different sound therapy options and identifying factors that may influence treatment outcomes and improve the quality of life of adults with hyperacusis. 

## 2. Materials and Methods

This scoping review follows Arksey and O’Malley’s (2005) [22] and the Joanna Briggs Institute (2020) [23] methodology framework for scoping reviews, as defined by the following stages:

- Stage 1—identifying the research question;
- Stage 2—inclusion/exclusion criteria and the location of relevant publications (identifying relevant studies);
- Stage 3—selection of relevant studies based on a screening of the abstract or reading the full journal/text (study selection);
- Stage 4—extraction and charting of data;
- Stage 5—collation and reporting of results;
- Stage 6—expert consultation (optional).

This scoping review has not been registered with PRISMA (Preferred Reporting Items for Systematic Reviews and Meta-analyses). The protocol for this scoping review was registered on the Open Science Framework on the 30 March 2024 (details within the Supplementary Materials section).

### 2.1. Research Question: Identification of the Research Question

What and how is sound therapy used to treat patients with hyperacusis?

### 2.2. Inclusion/Exclusion Criteria

The PCC (population/context/concept) framework was used to support the formulation of the research question and search strategy [24]. To be included, records were required to report studies with adults (≥18 years) experiencing hyperacusis and reporting the use of sound therapy devices (context/concept), including environmental sound, tabletop sound generators, sound generators, hearing aids/combination devices, and any other devices that were reported on. Studies published in any language were included, provided that they could be translated using google translate or Hospital Translation services. Studies published in the last 25 years were included, as there have been further advancements in hyperacusis research [25,26]. Peer-reviewed or grey literature, randomized control trials, non-randomized controlled trials, retrospective studies, case studies, and peer-reviewed books were included. Review articles including systematic reviews; studies focusing on tinnitus, misophonia, and phonophobia without hyperacusis; studies posted on social media or internet forums; and any sources reporting personal or expert opinion were excluded. 

### 2.3. Search Strategy

The search strategy, including all identified keywords and index terms, was adapted for each included database and/or information source (see Table 1). The electronic searches were completed in CINAHL (Cumulative Index to Nursing and Allied Health Literature), Cochrane Library, Medline via EBSCO, (Ipswich, MA, USA), Scopus, and PsycINFO. As an additional step, the reference lists of included sources of evidence were screened for additional studies. Electronic searches were completed in April 2024.

### 2.4. Study Selection

**Figure 1:**
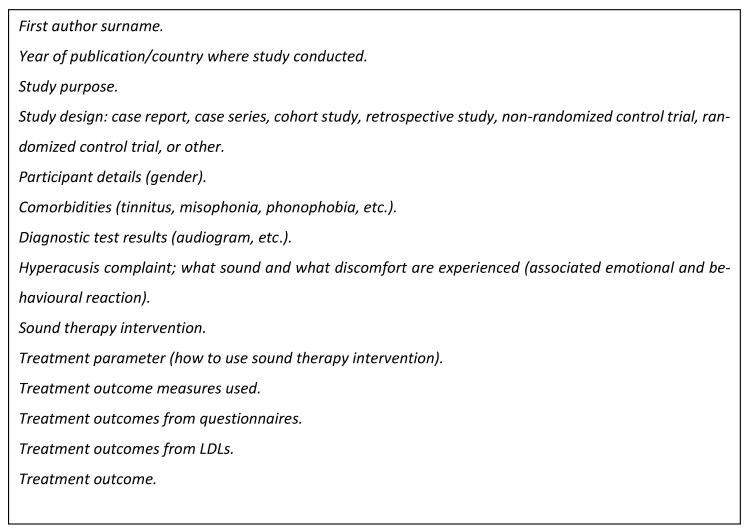
Data extraction fields.

Following the search, all identified records were collated and uploaded into Covidence software (version 1), and duplicates were removed. The titles and abstracts were then screened by two reviewers (N.K. and D.E.N.) for assessment against the inclusion criteria for the review. Potentially relevant sources were retrieved in full, and their record details were imported into Covidence (systematic review tool). Full text records were assessed in detail against the inclusion criteria by the lead author and one independent reviewer (N.K. and D.E.N.). Reasons for exclusion were recorded and reported in the PRISMA-SCR (Preferred Reporting Items for Systematic Reviews and Meta-analyses extension for scoping review flow diagram) [27] (Figure 1). K.F., an expert in this field, reviewed the included articles to support the assessment against the inclusion criteria. Any disagreements that arose between the reviewers were resolved through discussion or with an additional independent reviewer (K.F.).

### 2.5. Data Extraction

A data extraction form was developed, piloted, and subsequently modified following team discussions. Data were extracted by N.K. on study characteristics (e.g., gender, comorbidities, diagnostics tests hearing tests (audiograms), ULLs, hyperacusis complaint (troublesome sound and physical discomfort reported by patients), type of sound therapy, and how it was used and treatment outcomes) (Figure 1 shows data extraction fields). Quality assessments were not conducted, as this is an optional step in the methodology of scoping reviews. 

### 2.6. Data Collation

Data were collated and summarized descriptively to present current understandings of the recommended use of sound therapy.

## 3. Results

### 3.1. Study Selection 

**Figure 2:**
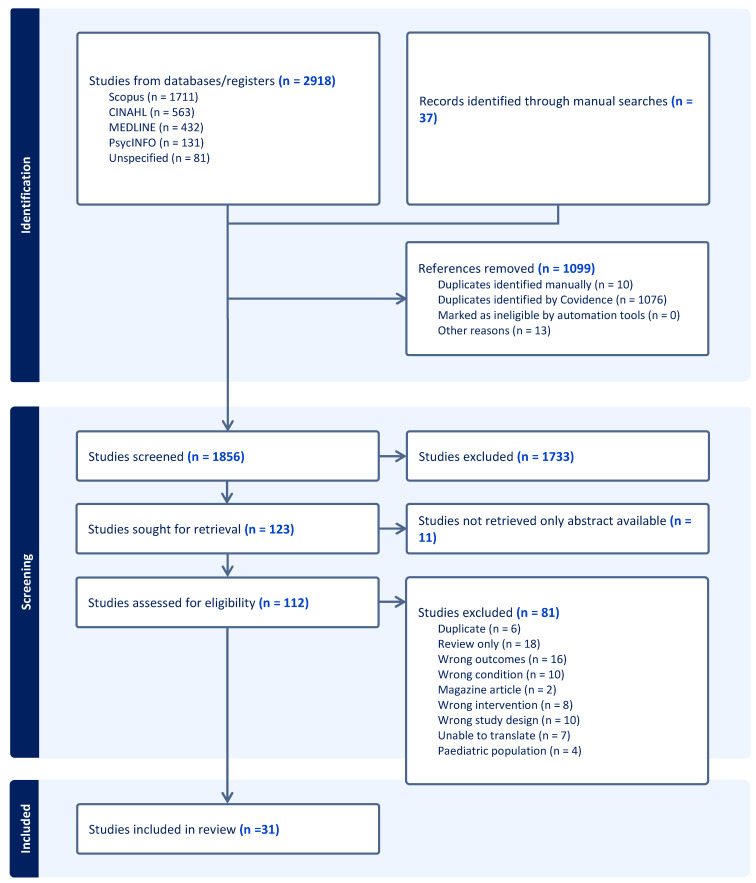
PRISMA-ScR flow diagram.

Electronic searches identified 2918 records. Through further manual searching, 37 records were identified. After duplicates were removed, the remaining 1856 records were screened by title and abstract by N.K. and D.E. Following this, 1733 were excluded due to not meeting the inclusion criteria (e.g., they did not report hyperacusis in adults or sound therapies), which resulted in 123 records being retrieved for full text screening. Of these, the full text for 11 records could not be retrieved, and of the remaining records, 81 did not meet the inclusion criteria (see Figure 2, PRISMA flow, for reasons for exclusion). A final list of 31 records was included in this review for data collection.

### 3.2. Study Characteristics 

Of the 31 articles, 23 were journal articles, 6 were conference papers, and 2 were articles included in published books and not available as separate published journal articles. The articles were published from 2000 to 2021. Fifteen articles reported studies from the USA, three studies were reported from the UK, twelve studies were reported from Europe, and one study was reported from the Republic of Korea. Of the included records, nine were case studies [28,29,30,31,32,33,34,35,36], four were cohort studies [37,38,39,40], five were RCTs [41,42,43,44,45], four were Non-RCTs [46,47,48,49], eight were retrospective studies [50,51,52,53,54,55,56,57], and one study was a comparative study [58].

### 3.3. Participant Characteristics

All of the studies reported hyperacusis as part of a symptom set with comorbidities [e.g., tinnitus]. However, there were two studies which did not specifically detail how many participants had hyperacusis [49,58]. There were 10 records that did not report the sex of participants [31,34,35,41,44,48,49,52,53,57,58]. The remaining studies reporting sex, excluding individual case studies, reported both female and male participants (n = 15) [29,30,31,37,38,39,42,43,46,47,50,51,54,55,56]).

### 3.4. Comorbidities

All records included patients with hyperacusis and at least one other comorbidity. Comorbidities included tinnitus (n = 26 [28,29,30,31,33,34,35,36,38,39,40,41,43,45,46,47,48,49,50,51,52,53,54,55,56,57,58]), hearing loss (n = 18 [28,29,30,34,36,37,38,41,42,43,44,46,47,48,54,55,56]) including conductive hearing loss (n = 1 [36]), misophonia (n = 5 [31,50,51,52,57]), phonophobia (n = 7 [31,32,48,51,52,53,57]), depression (n = 1 [32]), post-traumatic stress disorder (n = 1 [32]), and Williams syndrome (n = 1 [36]). 

### 3.5. Diagnostic Test Results

All records reported at least one diagnostic test result for their participants. Predominantly (87%), a hearing test (audiogram) and ULLs were used to diagnose hyperacusis [28,29,30,31,33,34,35,36,37,38,41,42,43,44,45,47,48,50,51,52,53,54,55,56,57]. One study [46] used multiple other tests including tympanometry, ARTs, and OAEs but did not complete ULLs. One study [58] only completed a hearing test (audiogram) and coupler measures on the devices (hearing aids with a sound therapy program/setting). One study [39] mentioned an ENT (Ear, Nose, and Throat) internal checkup but did not state what tests were conducted. 

### 3.6. Hyperacusis Complaint

There were three studies [29,50,56] which did not report or mention what the hyperacusis complaint was (what type of sound) or how it made the participants feel. The description of the experience of hyperacusis ranged from environmental sounds [28,29,34,37,42,58], hypersensitivity to sound [28,36,40,49], reduced sound tolerance [35,43,44,45,48,52,53,56,57], and discomfort to sound [28,34,51,55]. Several studies reported the emotional impact of hyperacusis, describing negative symptoms [32], reports of distress [33,43,47,54,57] and social isolation [28,39,52,55], and the inability to use hearing aids [41].

### 3.7. Sound Therapy Intervention (Intervention), How to Use Sound Therapy (Use), and the Outcome Measures Used

**Figure 3:**
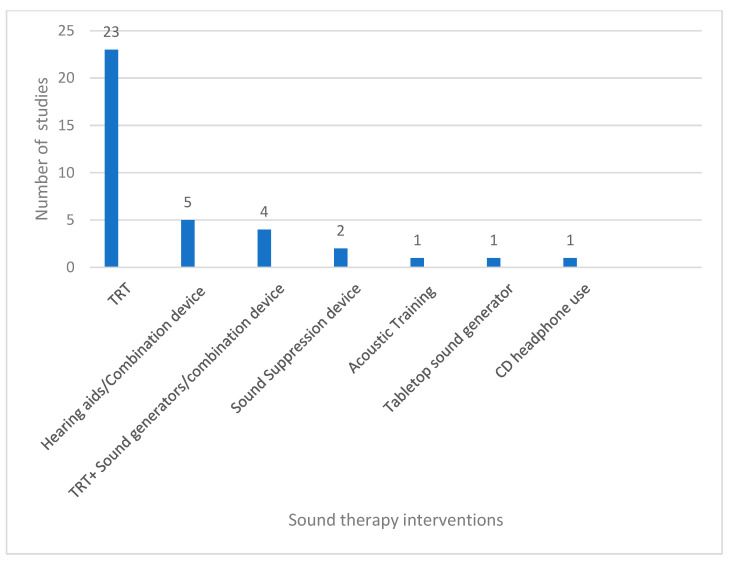
Sound therapy interventions.

Seven sound therapy interventions were described and a total of 19 outcome measures were used across the 31 studies, which are detailed below. Figure 3 shows the frequency of sound therapy interventions across the 31 studies. The most commonly used intervention was the TRT protocol. 

Intervention: TRT protocol: There were 23 studies reporting the use of the TRT protocol [28,30,31,32,33,34,39,40,41,43,44,45,47,48,50,51,52,53,54,55,56,57] (74%). 

Use: The remaining studies reporting the TRT protocol only specified sound generator use with a broadband signal for up to eight hours a day as a desensitization approach (this treatment approach also includes some directive counselling, which is not the focus of this scoping review). One study [47] reported Danalogic I Fit hearing aids (combination device) to be used as sound generators. 

The TRT protocol has a specific format, which follows a 36-item questionnaire/interview. Of the 36 questions, 11 questions (19–30) focus on decreased sound tolerance, and therefore, this is used as a basis for the partial diagnosis of hyperacusis. Following assessment (questionnaires and physiologic subjective testing (audiogram, ULLs)), patients are categorized into one of five domains within the TRT protocol to indicate whether they have non-bothersome tinnitus (0), bothersome tinnitus (1), bothersome tinnitus and hearing loss (2), hyperacusis and tinnitus (3), or tinnitus and misophonia/phonophobia (4). Treatment options include TRT counselling and sound therapy. Sound therapy involves advice on exposure to ambient enriched sound environments, advice about not blocking ears with ear plugs, the use of ear-level sound generators/hearing aids, or combination devices set at a level that is just audible with broadband noise, which are to be used all day for hyperacusis (>8 h). A tabletop sound generator is also advised at night in the TRT protocol. This was not always specified in the records included. The aims of the studies reporting implementing the TRT were mainly evaluating TRT for tinnitus and hyperacusis [28,29,30,31,32,33,34,35,38,39,40,41,42,43,44,45,46,47,48,50,51,52,53,54,55,56,57], and six studies also focused on gain changes in hearing-impaired individuals experiencing sound intolerance [30,31,40,41,51,56].

Outcomes: The outcomes used were a mixture of pre- and post-treatment ULLs [30,31,36,39,40,41,42,48,52,56], the dynamic range change [56], NU 6 word test [41,44], Contour 7 test of loudness perception [41,44], self-report questionnaires including the specially designed questionnaires [50,55], the GUFF questionnaire on hypersensitivity to sound [50], MASH [37,50], the Tinnitus Handicap Inventory (THI) [29,33,46,54,55,57], HQ [37,46,50,55], TRT interview questions [28,30,31,32,33,34,39,40,41,43,44,45,47,48,50,51,52,53,54,55,56,57], the TQ [43,47,49], the Tinnitus Reaction Questionnaire (TRQ) [32], BDI [33], the Hospital Anxiety and Depression Scale (HADs) [55], and subjective self-reporting [35]. All studies reported a positive change in symptoms.

Intervention: Acoustic Training: One study reported a form of acoustic training [36] (3%).

Use: One study reported the use of acoustic training [36] and used narrow band noise in a free field for one session every five days over thirty-five days. This was then followed by pure tones through headphones for three minutes of stimulation @60dBHL, followed by three minutes of rest, for six cycles of stimulation @60Dbhl. The final training moved to the use of Cocktail party sounds.

Outcomes: This study used LDLs/ULLs to assess hyperacusis post-intervention. The results indicated that LDLs improved post-intervention and an overall positive improvements in symptoms.

Intervention: CD player/headphone: One study reported the use of headphones/a CD player with an acoustic signal [37] (3%).

Use: One study reporting the use of headphones/a CD player [37] with an acoustic signal (this was derived from the audiogram with pure and weighted tones so that each participant had a specific signal to use based on the hearing test results) advised participants to listen daily for a few hours a day at an audible level.

Outcomes: This study [37] used loudness growth (LGOB), the Hyperacusis questionnaire, and the Multiple-Activity Scale for Hyperacusis (MASH) to assess hyperacusis. The results indicated that hyperacusis symptoms improved following this intervention, with decreased loudness growth and scores in both questionnaires.

Intervention: Phase-out device: One study reported an acoustic phase-out device as an intervention [49] (3%).

Use: The pure tone phase-out device [49] involved three in-office phase-out sessions that were thirty minutes long, and then, the participants were given the device to use at home for thirty minutes three days a week.

Outcomes: This study [49] conducted a pre-treatment hearing test (audiogram), ULLs, and Magnetic Resonance Imaging (MRI) and used the Visual Analogue Scale (VAS), Tinnitus Questionnaire (TQ), Hyperacusis questionnaire (HQ), and Beck Depression Scale (BDI). No improvement was seen in symptoms.

Intervention: Sound suppression device: Two studies reported the use of a sound suppression device. This included an electronic suppression device [38] and a Microtech refuge hyperacoustic instrument [28] (6%).

Use: The two studies reporting sound suppression devices [28,38] encouraged varied use. One advised using the device over a two-month period in uncomfortable situations, while 367 advised its use at all times or when required [28].

Outcomes: Conducted pre-treatment assessments were a hearing test (audiogram), ULLs, and reflex testing [28,38]. However, nine participants were unable to tolerate the reflex testing [38]. Additional pretesting procedures for study 37 were speech testing, tympanmetry, positional testing, evoked potentials/reflexes, Computer Tomography (CT) scan, and MRI [38]. The outcomes used were post-treatment ULLs [38] and self-reporting [28], and participants reported a positive reduction in symptoms [28,38].

Intervention: Tabletop bedside noise/sound generator: One study reported the use of a bedside tabletop noise generator as an intervention at night alongside the use of noise generators during the day (Siemens pure life open fit hearing aids) [51] (3%).

Use: One study specifically encouraged the use of a tabletop sound generator at night and the use of a combination device during the day [51].

Outcome: The outcome measurement tool used was the TRT questionnaire, and a positive change was reported in sound tolerance and dynamic range [51].

Intervention: Hearing aids/sound generators/combination devices: There were five studies referring to the use of hearing aids/sound generators with a sound therapy program [29,43,46,51,58] (16%).

Use: The studies reporting hearing aid/sound generator/combination devices reported the use of white noise/broadband noise continuously during the day [29,45,46,55,58], while four studies were implementing the TRT protocol [29,45,55,58]. One study also encouraged the use of a combination device (Siemens open fit hearing aids) during the day with a specific broadband noise shape (derived from psychoacoustic measurements that were specific to each participant) and encouraged using a tabletop sound generator at night [46].

Outcome: The outcomes used were varied and included the THI [29,46,55], HQ [46,55], TRT interview questions [45,55], VAS [29], HADs [55], and a specially designed questionnaire [55]. All studies reported a positive change in symptoms of hyperacusis.

No participant outcome measure: One study did not report the use of outcome measures, as the focus of the study was sound therapy output from a hearing aid device; therefore, the outcome was focused on coupler measures and hearing aid sound therapy output [58].

Table 2 details the different interventions and whether a negative or positive outcome was achieved.

## 4. Discussion

The aim of this scoping review was to establish the current use of sound therapy in adults with hyperacusis and identify any factors that may influence treatment. A brief summary was provided in the Section 3 to describe the interventions that have been reported in the literature, including acoustic training, headphone with CD use, sound suppression devices, tabletop sound generators, the use of hearing aids/sound generators/combination devices, and the TRT protocol.

Despite the large number of included records, all studies reported hyperacusis with comorbidities. This meant that all studies treated hyperacusis as a part of a symptom set. Therefore, the transferability of the review findings for the use of sound therapy in patients with hyperacusis as a standalone condition is limited. Many of the records also reported individual case studies or small numbers of participants. This review has highlighted the need for randomized controlled trials to evaluate the effectiveness of sound therapy interventions as treatments for hyperacusis populations.

Many of the studies used a hearing test (audiogram) and ULLs/LDLs to assess participants, supporting the use of physiological measures as reported by Baguley and colleagues in 2007 [7]. However, as part of this assessment process, the outcome measures used across all 31 records varied significantly. This variation across outcome measures leads to a difficulty in comparing the effectiveness of interventions when the outcome measure differs.

Surprisingly, there were no reports of sound therapy apps on mobile phones in the included papers, even though these are used for tinnitus [59] and are recommended by Tinnitus UK [60]. There is a lack of evidence supporting mobile app use for hyperacusis. There are limited descriptions of what sound enrichment entails or what types of sound to use for what length of time, which also shows a lack of evidence and impacts clinical practice advice for patients with hyperacusis. There were limited descriptions or advice on what manufacturers’ hearing aids/sound generators/combination devices to use or how to advise on the use of these with a sound therapy programme. The study that measured the output of a sound therapy programme [50] reported a higher output than expected, which could impact hyperacusis patients negatively. This calls for hearing aid manufacturers that provide combination devices to standardize the output of these devices, as otherwise, it could lead to variation in clinical practice and patient care. Although the outcomes of most of the studies were positive, treatment effects could not be purely attributed to sound therapy use alone, except in two studies [51,53]. This highlights the need for randomized controlled trials that evaluate the effectiveness of sound therapy interventions on its own. There is also a lack of evidence to support electronic attenuators or blocking ears due to the minimal numbers of studies reporting this.

The TRT protocol, as mentioned previously, includes a specific interview questionnaire and the use of sound generators with TRT counselling. The TRT protocol also encourages the use of a tabletop sound generator to be used at night. However, none of the studies specified or described the use of a tabletop sound generator. There was an implicit assumption that the TRT protocol was well understood in terms of what it entailed. Some of the TRT records did not specify the use of broadband noise as a sound therapy program on the hearing aids/sound generators/combination devices either, which again shows that an assumption was made that the reader would be aware of the TRT protocol. There was a high proportion of studies using the TRT protocol, and yet, there was limited information on its specific use, despite five randomized controlled trials evaluating the efficacy of TRT. There were only three studies conducted in the UK using the TRT protocol [21,27,41], and these were not randomized controlled trials. Most studies were conducted in the US and Europe, where healthcare is privatized, and training and education is different to that in the UK [61]. This may offer an explanation as to why there was a lack of description of the TRT protocol.

The clinical implications in the UK mean that clinicians may provide varying advice on sound therapy use, and this may impact patients’ symptoms in the long term, their quality of life, and their well-being [7,14]. The evidence for the use of sound therapy devices is conflicting and lacks systematic methodological evaluation. The TRT protocol is a guideline that has been developed in the US, and the TRT protocol has shown empirical evidence, with the use of sound therapy and counselling, indicating that it can be used as an intervention for hyperacusis with positive affects [29,41,42,49,55]. However, further randomized control methodologies and a clinician consensus are required to support its training and dissemination. There is a need to continue to explore and understand the experiences of hyperacusis and the management strategies that could be applied, as well as new ways or techniques that could be used for sound therapy interventions.

This scoping review provides valuable insights into hyperacusis management with the use of sound therapy. However, several limitations should be considered regarding the data included. Many studies were individual case studies or retrospective studies, indicating limited information about sound therapy use and its efficacy. The lack of randomized controlled trials reduced the ability to compare the effective use of sound therapy interventions without bias. The lack of standardized verified outcome measures limits data for comparison of pre- and post-treatment outcomes within individual studies and crossdata examination. Many of the studies did not focus on hyperacusis management, as hyperacusis was part of a symptom set; therefore, an improvement in symptoms may not be reflective of a hyperacusis population. Some studies relied on self-reported data, which may have introduced response bias by participants either overestimating or underestimating their improvement. Also, many different outcome measures were used, which cannot be compared due to impacting the external validity. Future research should focus on hyperacusis and sound therapy use using a methodical RCT design and aim to reduce these limitations. This has also been stated in previous research [2].

## 5. Conclusions

There is limited evidence supporting the use of sound therapy for patients with hyperacusis. There is a further lack of evidence describing specific intervention parameters. Despite frequent use of the TRT protocol, further randomized controlled trials are required to determine the protocol’s effectiveness in treating hyperacusis. Future research should look to explore the use of interventions including sound enrichment, acoustic training, headphone CD use, tabletop sound generators, and hearing aids/sound generators/combination devices. Finally, a consensus on the current interventions used (what and how) within the UK is warranted due to the gaps in knowledge. This can be optimized by producing high-quality research with use of randomized controlled trials and with clinician Delphi consensus, which could inform clinical practice in the UK.

## Figures and Tables

**Table 1:** Search term strategies for hyperacusis sound therapy intervention. CINAHL = Cumulative Index to Nursing and Allied Health Literature; Medline (EBSCO); Psychinfo; Cochrane Library; Scopus. (∗ = And).

Search Terms	Search Engine
Hyperacusis AND [Sound therapy ∗ OR white noise generators ∗ OR Sound generators ∗ OR Treatment with sound ∗ OR Graded exposure ∗ Desensitization to sound	CINAHL, Medline (EBSCO), Psychinfo, Cochrane Library and Scopus
Sound sensitivity/Intolerance to sound/Reduced gain to sound/Hypersensitivity to sound/Decreased sound tolerance = combined as above	CINAHL, Medline (EBSCO), Psychinfo, Cochrane Library and Scopus

**Table 2:** Outcomes of interventions.

Intervention	Outcome
Acoustic training	Positive—Improvement seen in symptoms/tolerance
Headphone/CD player	Positive—Improvement seen in symptoms/tolerance
Phase-out device	Negative—No improvement seen in symptoms/tolerance
Sound suppression devices	Positive—Improvement seen in symptoms/tolerance
TRT protocol	Positive—Improvement seen in symptoms/tolerance
Tabletop sound generators	Positive—Improvement seen in symptoms/tolerance
Hearing aids/sound generators/combination devices	Positive—Improvement seen in symptoms/tolerance

## Data Availability

Dataset available on request from the authors. The raw data supporting the conclusions of this article will be made available by the lead author on request.

## Supplementary Materials

The following supporting information can be downloaded at: https://www.mdpi.com/article/10.3390/brainsci14080797/s1, What are the current recommendations on how to use sound therapy to treat adult patients diagnosed with Hyperacusis? A scoping review; The protocol for this scoping review was registered on the Open Science Framework on the 30 March 2024. This can be accessed at https://osf.io/6xkwe (Last accessed 4 August 2024). The data extraction supplemental information can be accessed upon request to the lead author via email.
